# Highly Efficient Photocatalysis Towards Synthesis of Crystalline Hydrothermal Carbonation Carbon

**DOI:** 10.3390/molecules31091421

**Published:** 2026-04-25

**Authors:** Xunxian Chen, Yu Luo, Zihang Zhang, Yingming Chen, Zhen Wan

**Affiliations:** 1 Foshan Water Industry Group Co., Foshan 528000, China; 13827722820@sohu.com; 2 Key Laboratory of Catalysis and Materials Science of the State Ethnic Affairs Commission and Ministry of Education, College of Resources and Environmental Science, South-Central Minzu University, Wuhan 430074, China; cy921127@outlook.com (Y.L.); 2025120752@mail.scuec.edu.cn (Z.Z.); chenym@mail.scuec.edu.cn (Y.C.)

**Keywords:** crystalline HTCC, cellulose, microcystin-LR, Cr(VI), hydrogen radicals

## Abstract

As a novel organic semiconductor derived from biomass, hydrothermal carbonation carbon (HTCC) usually exhibits an amorphous structure due to its well-recognized formation pathway based on 5-hydroxymethylfurfural (HMF), which impedes charge transfer and consequently restricts the photocatalytic activity. Herein, we report a crystalline HTCC photocatalyst produced via an unusual synthesis route applied to cellulose in the presence of an oxidant. Notably, the crystalline structure of cellulose was retained and became highly aromatized during the process, leading to significantly enhanced charge transfer efficiency and an increased density of active sites. Moreover, unlike other reported HTCC photocatalysis, the highly active hydrogen radicals (H•) were identified as the dominant active species governing photocatalytic Cr(VI) reduction over crystalline HTCC. As a result, this crystalline HTCC exhibited dramatically enhanced photocatalytic removal efficiencies of Cr(VI) and microcystin-LR (MC-LR) due to the highly efficient charge transfer, abundant active sites as well as highly active hydrogen radicals.

## 1. Introduction

As a type of biomass-derived carbon photocatalyst, hydrothermal carbonation carbon (HTCC) has attracted growing attention recently [1,2]. Generally speaking, HTCC is attractive for the following reasons: (i) The HTCC can be produced by treatment of agricultural residues (e.g., reed) or animal wastes (e.g., sheep dung) that contain large amounts of biomass. Valorizing such wastes into useful products is of great significance to environmental sustainability [3,4]. (ii) Unlike metal-based catalysts that are characterized by high cost and a potential for detrimental metal leaching, the HTCC is a “cheap” alternative and would not cause secondary pollution [4]. (iii) It is easy to modulate the surface chemical properties of HTCC by grafting it with organic functional groups which are critical to the activity and selectivity in reactions [5,6]. However, since the formation of HTCC typically involves a dehydration step in which precursors are initially converted into HMF, the resulting HTCC materials are often composed of discrete polyfuran units. These units exhibit an amorphous structure, leading to inefficient photocatalytic performance due to limited charge transfer and poor conductivity [1,7]. Therefore, in previous studies of HTCC-based photocatalysis, the HTCC materials were usually combined with other semiconductors or metals [8,9,10].

**Figure 1:**
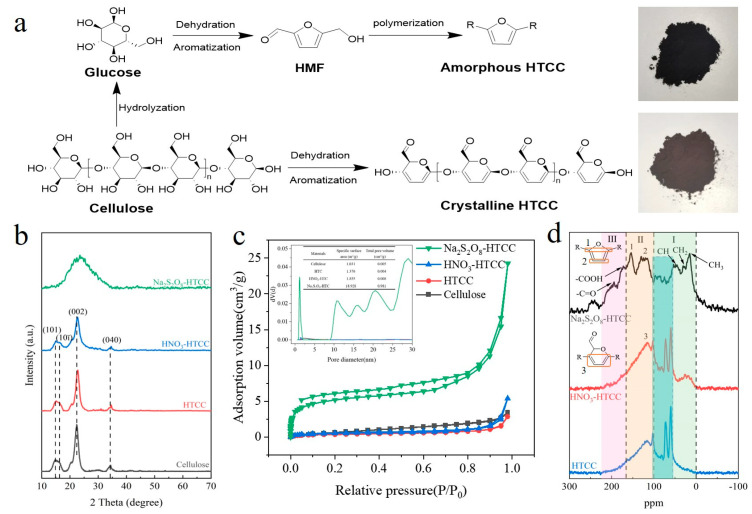
(**a**) Schematic synthesis process of crystalline and amorphous HTCC produced by cellulose. (**b**) XRD patterns, (**c**) N_2_ isotherm adsorption/desorption curves and (**d**) ^13^C solid-state CP-MAS NMR spectra of HTCC photocatalysts.

Cellulose serves as a prevalent precursor of HTCC, which offers an alternative approach to address this issue owing to its inherent stability of a crystalline structure [11]. Unlike the conventional HMF-mediated formation pathway, cellulose molecules underwent direct dehydration and aromatization during the low-temperature hydrothermal process. This alternative route preserved the crystalline structure of cellulose [12,13], albeit resulting in a poor aromatization level that limits its potential applications (Figure 1a). Therefore, enhancing the aromatization level of cellulose-based HTCC while retaining its crystalline structure represents a viable approach for achieving an efficient HTCC photocatalyst. In this study, HTCC photocatalyst with a well-aromatized crystalline structure was produced via the nitric acid-assisted hydrothermal treatment of cellulose. Due to the efficient charge transfer and abundant active sites, the photocatalytic performances of the crystalline HTCC for the removal of Cr(VI) and MC-LR were dramatically improved. Moreover, unlike other reported HTCC photocatalysts, we confirmed that the hydrogen radicals (H•) dominated the photocatalytic Cr(VI) reduction processes of this crystalline HTCC.

Herein, HTCC photocatalysts generated by hydrothermal treatment assisted with nitric acid and sodium persulfate were denoted as HNO_3_-HTCC and Na_2_S_2_O_8_-HTCC, respectively, while the oxidant-free sample was directly denoted as HTCC.

## 2. Results and Discussion

We first investigated the crystalline structure of as-prepared HTCC photocatalysts by XRD (Figure 1b). Identical to the pattern of cellulose, HTCC and HNO_3_-HTCC exhibited multiple diffraction peaks associated with the carbon fiber, indicating the presence of a crystalline structure [3,4]. This crystalline structure remained intact even with an increased concentration of added nitric acid (Figure S1). On the contrary, the Na_2_S_2_O_8_-HTCC only showed a broad diffraction peak centered at 22.1°, confirming its amorphous structure. This is similar to the results of HTCC derived from glucose [14], suggesting that the crystalline structure of cellulose was destroyed under robust oxidation of sodium persulfate. N_2_ adsorption–desorption isotherms further confirmed this result. In Figure 1c, the HTCC, HNO_3_-HTCC and original cellulose all displayed a type II isotherm, indicating that these materials are non-porous. From the inset, the BET and total pore volume of these samples exhibited minimal variations, which further corroborated the XRD analysis that the HTCC and HNO_3_-HTCC retained the fiber structure of the original cellulose. By contrast, the Na_2_S_2_O_8_-HTCC showed a type IV isotherm with a hysteresis loop of H4 in medium region (0.2–0.8 P/P_0_), which can be ascribed to a mesoporous structure. Moreover, its BET and total pore volume value were dramatically increased, which is strikingly different in from that of the HTCC and HNO_3_-HTCC, further confirming its amorphous structure. The detailed chemical structures of HTCC photocatalysts were demonstrated by the ^13^C solid-state CP-MAS NMR spectra. In Figure 1d, a primary qualitative attribution based on the literature was proposed for each spectral domain [15,16]. region I (0–100 ppm) is characteristic of sp^3^-carbon atoms. The broad distribution in 0–55 ppm was attributed to the CH_x_ (x = 1–3) sites from the hydrolyzed by-products. Particularly, unlike Na_2_S_2_O_8_-HTCC, the HTCC and HNO_3_-HTCC exhibited evident peaks in the 60–105 ppm region, in which the peaks at 72.2 and 102.6 ppm corresponded to C2/C5 and C1 sites of cellulose, respectively, while the peak at 60.2 ppm represented C-OH groups of the hydrolyzed by-products [17]. Other characteristics of original cellulose are not obvious, indicating the aromatization of cellulose structure. Region II (100–160 ppm) is characteristic of sp^2^-carbon atoms. In spectrum of Na_2_S_2_O_8_-HTCC, the peaks at 152 ppm and 115–127 ppm corresponded to O–C=C and C=C–C bonds of the polyfuran moieties [1,3,4]. However, due to the p–π conjugation caused by elevated oxygen content (Table S2), the HTCC and HNO_3_-HTCC exhibited the broad peak shifted towards higher magnetic fields compared with the Na_2_S_2_O_8_-HTCC. By comparing the proportions of region II, it is clear that the HNO_3_-HTCC exhibited higher aromatization level than HTCC, which might be ascribed to the further dehydration of cellulose.

**Figure 2:**
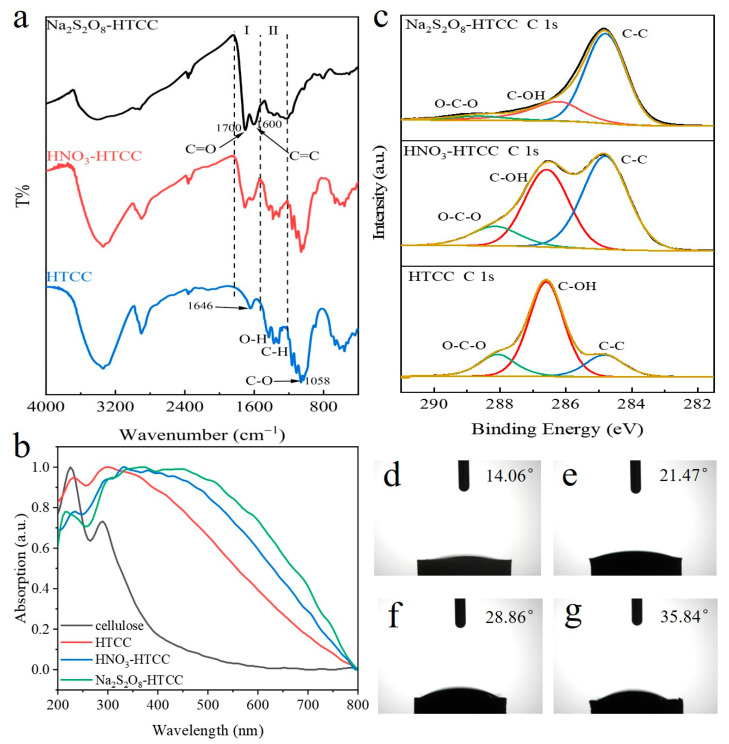
(**a**) FT-IR, (**b**) XPS C 1s and (**c**) UV-vis diffuse reflection spectra of HTCC products. Surface contact angle measurements of (**d**) cellulose, (**e**) HTCC, (**f**) HNO_3_-HTCC and (**g**) Na_2_S_2_O_8_-HTCC.

**Figure 3:**
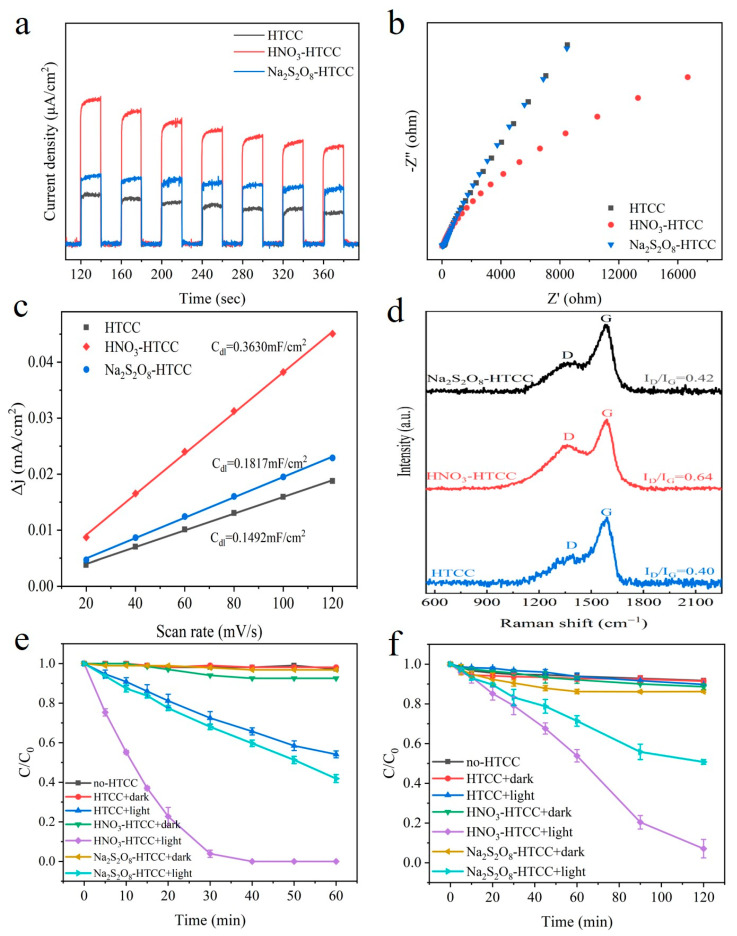
(**a**) Photocurrent responses, (**b**) electrochemical impedance spectra, (**c**) C_dl_ values and (**d**) Raman spectra of HTCC photocatalysts. Time profiles for the photocatalytic Cr(VI) reduction (**e**) and MC-LR degradation (**f**) over HTCC photocatalysts under irradiation.

FT-IR experiments were then performed to further study the aromatization level of HTCC photocatalysts (Figure 2a, Figures S2 and S3). As shown in Figure 2a, both the HTCC and HNO_3_-HTCC exhibited a distinct wide band at 1058 cm^−1^, which can be assigned to the C–O stretching of cellulose. Combined with the XRD analysis results, this finding further indicated the existence of a crystalline structure. The bands at region II (1500–1220 cm^−1^) could be allotted to the aliphatic C–H and O–H bending modes while the shoulder bands at region I (1835–1540 cm^−1^) could be assigned to the sp^2^ hybridized C=C (1600 cm^−1^) and C=O (1700 cm^−1^) stretching, respectively [1,14]. Meanwhile, the HTCC showed the characteristic peak of adsorbed water at 1646 cm^−1^ which covered other bands in region I. We assessed the aromatization level of different HTCC samples by comparing the ratio of region I/II (Table S1). Apparently, compared with that of HTCC (0.116), the ratios of HNO_3_-HTCC (0.787) and Na_2_S_2_O_8_-HTCC (1.116) are significantly higher, which indicated a marked improvement in the aromatization level. UV-vis diffuse reflection spectra were also performed to study the aromatization level of HTCC samples (Figure 2b and Figure S4). In Figure 2b, unlike original cellulose, all HTCC photocatalysts exhibited intrinsic semiconductor-like absorption in the wavelength range of 300–800 nm, representing the excited π electrons in the sp^2^ hybridized structures [18,19]. Assisted by the oxidants in hydrothermal treatment, the adsorption of HNO_3_-HTCC and Na_2_S_2_O_8_-HTCC displayed an obvious red shift compared with that of the HTCC, indicating their higher aromatization level, in agreement with the FTIR results. Additionally, the intramolecular dehydration level is also a crucial signal to assess the aromatization level. In XPS C 1s spectra (Figure 2c), three peaks assigned to C–C, C–OH and O–C–O bonds could be observed for all HTCC photocatalysts from lower binding energy to higher [3,4]. Compared with that of HTCC, the peaks of C–OH bond in HNO_3_-HTCC and Na_2_S_2_O_8_-HTCC both decreased, consistent with the results in O 1s spectra (Figure S5), indicating the enhancement of dehydration level. Figure 2d–g showed the surface contact angle measurements of original cellulose and three HTCC photocatalysts, in which the surface contact angle (CA) of the above samples gradually increased. Compared with that of the original cellulose and HTCC, the declined hydrophilicity of HNO_3_-HTCC could be assigned to the reinforced dehydration in aromatization process, which is also evident in the thermogravimetry and elemental analysis (Figure S6 and Table S2). Through the above results, compared with the HTCC with poor aromatization level and Na_2_S_2_O_8_-HTCC with amorphous structure, we confirmed that the HNO_3_-HTCC exhibited superior properties which maintained the crystalline structure derived from cellulose while improving its aromatization level. We inferred that this well-aromatized crystalline structure could potentially enhance the charge transfer efficiency. To support this assertion, transient photocurrent responses (Figure 3a) and electrochemical impedance spectra (EIS, Figure 3b) measurements on HTCC photocatalysts were performed. As shown in Figure 3a, the HNO_3_-HTCC exhibited significantly stronger photocurrent intensity than that of the amorphous Na_2_S_2_O_8_-HTCC, indicating its crystalline structure dramatically improved the charge transfer efficiency. Moreover, the photocurrent intensity of HNO_3_-HTCC was also about three times higher than that of the HTCC, which could be attributed to the improvement of aromatization level. In Figure 3b, the EIS plot of HNO_3_-HTCC exhibited a smaller arc radius than HTCC and Na_2_S_2_O_8_-HTCC did, contributing to the faster transfer of photogenerated carriers, which is consistent with above photocurrent responses results [20].

Meanwhile, we confirmed that the surface-active sites are also closely associated with the well-aromatized crystalline structure mentioned above. Herein, the electrochemical active surface area (ECSA) was used to identify the concentration of active sites of HTCC photocatalysts. To this end, cyclic voltammetry (CV) tests were conducted and double-layer capacitance (C_dl_) was used to reflect the ECSA of HTCC samples [21]. Based on the charging current density (ΔJ) obtained in Figure S7, the C_dl_ values were determined by fitting the scatter plot of ΔJ versus scan rate in Figure 3c. The C_dl_ value of HNO_3_-HTCC is 0.3630 mF‧cm^−2^, which was significantly higher than that of the Na_2_S_2_O_8_-HTCC (0.1817 mF‧cm^−2^) and HTCC (0.1492 mF‧cm^−2^), indicating its larger ECSA value and more active sites for photocatalysis. Raman spectra measurements are consistent with the ECSA results. As shown in Figure 3d, all HTCC photocatalysts exhibited the D band at 1332 cm^−1^ and G band at 1571 cm^−1^, corresponding to the vibrations of disordered carbon and sp^2^ hybridized carbon, respectively [22]. The ratio of D band to G band (I_D_/I_G_) can reflect the concentration of defects, which usually serve as active sites in the reaction. It is observed that the I_D_/I_G_ of HNO_3_-HTCC (0.64) was significantly higher than that of the Na_2_S_2_O_8_-HTCC (0.42) and HTCC (0.40), indicating the existence of more active sites in the structure of HNO_3_-HTCC.

**Figure 4:**
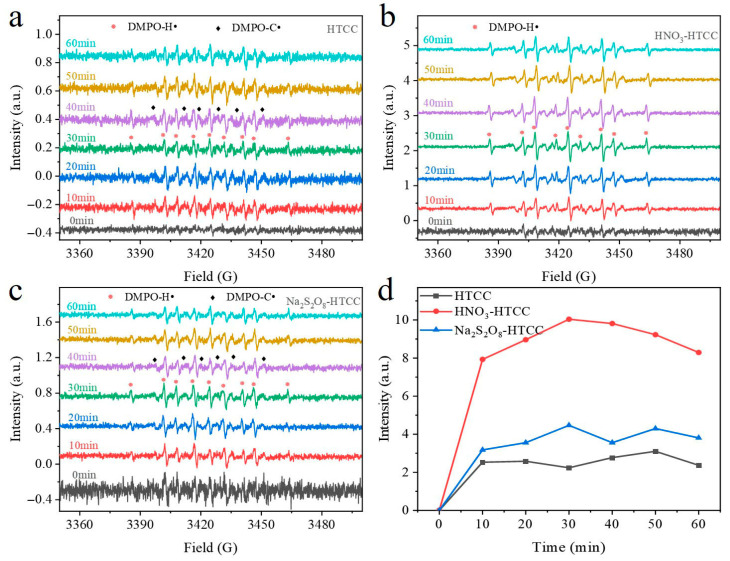
ESR spectra of DMPO spin adducts in the presence of (**a**) HTCC, (**b**) HNO_3_-HTCC and (**c**) Na_2_S_2_O_8_-HTCC after irradiation for different time periods. (**d**) ESR signal intensity of DMPO-H• for HTCC photocatalysts.

After exploring the structural features, all HTCC photocatalysts were applied to the photocatalytic reductive removal of Cr(VI). As shown in Figure 3e, the photolysis of Cr(VI) was negligible in the absence of photocatalyst, and the adsorption removal (if any) of Cr(VI) in the dark by all HTCC photocatalysts was also minimal. Under irradiation, HTCC and Na_2_S_2_O_8_-HTCC displayed weak activities, yielding the Cr(VI) removal of 45.9% and 58.2% within 60 min, respectively. However, the HNO_3_-HTCC exhibited appreciable activity such that the removal of Cr(VI) was nearly complete within 30 min and the rate constant (k) was 7.0 and 5.2 times higher than that of the HTCC and Na_2_S_2_O_8_-HTCC, respectively (Figure S8). Undoubtedly, the crystalline structure derived from cellulose played a pivotal role in enhancing the photocatalytic performance of HNO_3_-HTCC. This is evident from the fact that amorphous HTCC prepared by other sources of biomass (Figures S9 and S10), even with the addition of nitric acid during the preparation to enhance aromatization level, did not exhibit significant improvement in photocatalysis. The photocatalytic reduction in Cr(VI) by HNO_3_-HTCC under various pH conditions, as well as that over HNO_3_-HTCC modified with different nitric acid concentrations, were investigated, and the corresponding results were presented in Figures S11 and S12. To understand the mechanism of photocatalytic reduction process, the role of reactive species induced by photogenerated electrons was determined by ESR measurements and scavenger quenching experiments. As shown in Figure 4a–c, all HTCC photocatalysts displayed obvious ESR signals assigned to carbon-centered radicals (C•)–DMPO or hydrogen radical (H•)–DMPO adducts [23]. Typically, DMPO–C• adduct displays six-fold peaks line with a pattern of 1:1:1:1:1:1 (a^H^ = 24.6 G, a^N^ = 15.6 G, g = 2.0056) while the DMPO–H• adduct shows a 1:1:2:1:2:1:2:1:1 line shape (a^H^ = 15.5 G, a^N^ = 20.6 G, g = 2.0050) [24,25]. DMPO–C• was derived from the persistent free radicals (PFRs, Figure S13) and the DMPO–H• was generated by photogenerated electrons and protons [26]. Apparently, unlike amorphous Na_2_S_2_O_8_-HTCC and poor-aromatized HTCC, the HNO_3_-HTCC with well-aromatized crystalline structure exhibited intensive signal of DMPO–H• adducts during the irradiation (Figure 4d). Considering the absence of superoxide anion (•O_2_^−^) (Figure S14), it can be inferred that the reduction reactions of HNO_3_-HTCC were primarily driven by this highly active H• species. Furthermore, in scavenger quenching experiments, the monochloroacetic acid (MCAA) and nitrite were used to quench H• species and hydrated electrons (e_aq_^−^). As shown in Table S3, unlike nitrite (Ke_aq_^−^ = 3.5 × 10^9^ M^−1^ s^−1^, K_H•_ = 7.1 × 10^8^ M^−1^ s^−1^), MCAA has a much higher reaction rate with e_aq_^−^ (Ke_aq_^−^ = 6.9 × 10^9^ M^−1^ s^−1^, K_H•_ = 6.5 × 10^3^ M^−1^ s^−1^) than that with H• [23]. In Figure S15, the nitrite displayed more intensive inhibition effect than MCAA in the reactions of HNO_3_-HTCC and Na_2_S_2_O_8_-HTCC, indicating the H• species dominated these reactions. Compared with amorphous Na_2_S_2_O_8_-HTCC, the well-aromatized crystalline structure of HNO_3_-HTCC favored charge transfer efficiency and thus resulted in a high concentration of H• species generation. The as-prepared HTCC photocatalysts were also applied to the photocatalytic degradation of MC-LR. As shown in Figure 3f, compared with Na_2_S_2_O_8_-HTCC and HTCC, the HNO_3_-HTCC exhibited significantly enhanced photocatalytic performance, achieving 94% degradation of MC-LR within 120 min, which also can be ascribed to its well-aromatized crystalline structure. Moreover, the cyclic tests of HNO3-HTCC (Figure S16) exhibited great stability of the as-prepared HTCC photocatalysts.

## 3. Materials and Methods

### 3.1. Chemical Reagents

Nafion was obtained from Sigma Aldrich (St. Louis, MO, USA). 5,5-Dimethyl-1-pyrroline·N-oxide (DMPO) was provided by Aladdin Biological Technology Co., Ltd. (Xi’an, China). Flour and starch were purchased from COFCO Corporation (Beijing, China). Deionized water was obtained from Wahaha Group Co., Ltd. (Hangzhou, China). Microcrystalline cellulose, D-glucose and other chemical reagents were purchased from Sinopharm Chemical Reagent (Shanghai, China). All chemicals used in this work were used without any purification.

### 3.2. Synthesis of HTCC

HTCC was synthesized via a hydrothermal method. Typically, 6.0 g of cellulose ((C_6_H_10_O_5_)_n_) was dispersed into 60 mL of deionized water to form a suspension. After intensive stirring for 20 min, the suspension was transferred into a stainless steel Teflon-lined autoclave of 100 mL capacity and heated at 180 °C for 12 h. After the reaction, the samples were collected via vacuum filtration and dried in an oven at 60 °C for 12 h. Besides cellulose, glucose, starch and flour were also used to prepare the HTCC photocatalysts via the same method as a control.

### 3.3. Synthesis of HNO_3_-HTCC and Na_2_S_2_O_8_-HTCC

HNO_3_-HTCC and Na_2_S_2_O_8_-HTCC were synthesized via oxidant-assisted hydrothermal method. Typically, 6.0 g of cellulose was dispersed into 60 mL of nitric acid or sodium persulfate solutions (0.8 M). Subsequently, after intensive stirring for 20 min, the above suspensions were transferred into a stainless steel Teflon-lined autoclave of 100 mL capacity and heated at 180 °C for 12 h. The photocatalysts were collected via vacuum filtration and dried in an oven at 60 °C for 12 h. Other HTCC photocatalysts from different precursors (glucose, starch and flour) were also prepared via the nitric acid assisted hydrothermal treatment as a contrast.

### 3.4. Characterization and Testing

X-ray diffraction (XRD) was performed on a D8 ADVANCE diffractometer with Cu K_α_ radiation from Bruker Daltonics GmbH & Co., located in Bremen, Germany. The Brunauer–Emmett–Teller (BET) data were measured by N_2_ adsorption–desorption isotherms obtained at 77 K on an Autosorb-iQ (Quantachrome, Boynton Beach, FL, USA) after a vacuum degassing process overnight. ^13^C solid-state magic angle spinning (MAS) nuclear magnetic resonance (NMR) experiments were performed on JEOL JNM-ECZ600R 150 MHz spectrometer using the 3.2 mm zirconia rotors as sample holders spinning at MAS rate of 10 kHz (Hitachi, Tokyo, Japan). Raman spectra were carried out using a DXR Raman microscopy system (Thermo Scientific, Waltham, MA, USA) with an excitation wavelength of 532 nm. FT-IR spectra were recorded using an INVENIO-R spectrometer, which is equipped with an MCT detector cooled by liquid N_2_ (Bruker Daltonics, Bremen, Germany). X-ray photoelectron spectroscopy (XPS) analyses were carried out on a Thermo ESCA-Lab250Xi spectrometer with monochromatic Al Ka radiation while all the binding energies were calibrated by carbon (C1s 284.8 eV). UV-vis diffuse reflectance spectra (DRS) were detected by a Shimadzu UV2600 spectrophotometer (Shimadzu, Kyoto, Japan), using BaSO_4_ as the reflectance standard. Surface contact angle (CA) measurements were carried out on JC2000D1 machine (Powereach, Shanghai, China). A single droplet volume for each measurement was 2 μL. The content of the elements (C, H, O, N) of samples was analyzed using a Unicube elemental analyser (EA) while the thermogravimetry analysis was performed using a Shimadzu TGA-60H thermal analyzer. The atmosphere was N_2_ and the heating rate was 10 °C/min.

The formed reactive species, including •H or O_2_^•−^ species, were investigated through ESR spectrometer (Bruker ESR EMX nano, Bruker, Billerica, MA, USA) taking DMPO as an adduct agent. The samples were illuminated for given time by a 300 W Xe lamp, immediately mixed with DMPO solution (20 μL) and then measured [27,28]. Typical parameters for ESR measurements were as follows: the sweep width was 200 G, the modulation amplitude was 1.00 G, and the microwave power was set specifically at 10 mW. Persistent free radicals (PFRs) were also identified by ESR measurements [29,30]. Photocurrent, electrochemical impedance spectroscopy (EIS) and cyclic voltammetry (CV) curves were acquired on a CHI6601e electrochemical workstation (CH Instruments, Shanghai, China) with a standard three-electrode cell, where the sample coated ITO electrode was used as the working electrode, a Pt electrode as auxiliary electrode, and a saturated-potassium-chloride silver chloride electrode as reference. A 350 W xenon lamp was served as the light source and Na_2_SO_4_ (0.5 M) aqueous solution was used as the electrolyte. In the potential range of 0.1V, cyclic voltammetry test of the HTCC samples were carried out under different sweep speeds (20, 40, 60, 80, 100 and 120 mV · s^−1^). Then absolute value of current density difference at the median potential under each sweep speed could be calculated according to the CV curves. The absolute value of current density difference at the median potential and the sweep speed could be linearly fitted to obtain the slope of the fitting curve. The slope is the double-layer capacitance (C_dI_) [21,31].

### 3.5. Photocatalytic Experiments

The photocatalytic reduction of aqueous Cr(VI) by the as-prepared HTCC samples was conducted under a 300 W Xe lamp irradiation and room temperature which was kept by a circulating water bath. Briefly, aqueous Cr(VI) solution (20 mg L^−1^, pH 2.5) was prepared by diluting the K_2_Cr_2_O_7_ stock solution with deionized water. The HTCC sample (10 mg) was added into aqueous Cr(VI) solution (50 mL), followed by stirring for 30 min in the dark to reach the adsorption equilibrium. Then the suspension was illuminated by a 300 W Xe lamp equipped with a 330 nm cutoff filter. During the photocatalytic reaction, 0.5 mL of the reaction suspension was sampled at specified time intervals to determine the concentration of Cr(VI) using the standard diphenylcarbazide method [32]. The cyclic test was conducted under identical experimental conditions to those employed in the photocatalytic reduction of Cr(VI). Prior to each cycle, the recovered samples, including attached particles in the centrifuge tube and remaining sediment in the container, were collected and dried for 6 h. In addition, the following standard curve for quantifying the concentration of Cr(VI) was generated by analyzing the solutions containing Cr(VI) at concentrations ranging from 5 to 50 mg/L.

The photocatalytic degradation of microcystin-LR (MC-LR) by the as-prepared HTCC samples was conducted under identical experimental conditions to those employed in the photocatalytic reduction of Cr(VI) except for the concentrations of MC-LR (0.5 mg/L). The concentration of MC-LR was analyzed by a high-performance liquid chromatography (HPLC, Dionex, U3000, DIONEX, Sunnyvale, CA, USA). For the HPLC analysis, the injection volume to a diamonsil C-18 column was 50 μL. The mobile phase in isocratic mode with a flow rate of 1.0 mL/min was a mixture of trifluoroacetic acid aqueous solution and acetonitrile at a volume ratio of 13:7. The MC-LR was measured at 238 nm. The photocatalytic performances were evaluated using time profiles of C/C_0_, where C is the concentration of the substrates at each irradiation time, and C_0_ represents the concentration at the equilibrium point before irradiation, respectively. The apparent rate constant was calculated by the equation of k = In(C_0_/C)/t [33].

## 4. Conclusions

In summary, we proposed a strategic approach to acquire a highly efficient HTCC photocatalyst by constructing a well-aromatized crystalline structure based on cellulose. The introduction of nitric acid during the hydrothermal treatment was found to promote the aromatization of HTCC photocatalyst while preserving its original crystalline structure. This well-aromatized crystalline structure derived from cellulose has a critical impact on HTCC photocatalysis by enhancing the charge transfer efficiency and generating more abundant active sites. By contrast, the original HTCC, amorphous Na_2_S_2_O_8_-HTCC and HTCC prepared by other precursors all exhibited weak photocatalytic performances. Moreover, for the first time, we presented evidence that the highly active hydrogen radicals played a dominant role in the photocatalytic reduction of HTCC photocatalysis.

## Data Availability

The data presented in this study are available upon request from the corresponding author.

## Supplementary Materials

The following supporting information can be downloaded at: https://www.mdpi.com/article/10.3390/molecules31091421/s1, Figure S1: XRD patterns of HNO_3_-HTCC prepared by hydrothermal treatment added with different concentrations of nitric acid; Figure S2: FT-IR spectrum of original cellulose; Figure S3: FT-IR spectra of HNO_3_-HTCC prepared by hydrothermal treatment added with different concentrations of nitric acid; Figure S4: The Converted Kubelka-Munk plots of HTCC, HNO_3_-HTCC and Na_2_S_2_O_8_-HTCC; Figure S5: XPS O 1s spectra of the (a) HTCC, (b) HNO_3_-HTCC and (c) Na_2_S_2_O_8_-HTCC; Figure S6: Thermogravimetry curves of HTCC, HNO_3_-HTCC and Na_2_S_2_O_8_-HTCC; Figure S7: CV curves of (a) HTCC, (b) HNO_3_-HTCC and (c) Na_2_S_2_O_8_-HTCC; Figure S8: The apparent rate constants of HTCC, HNO_3_-HTCC and Na_2_S_2_O_8_-HTCC in the photocatalytic Cr(VI) reduction; Figure S9: FT-IR spectra of (a) HTCC and (b) HNO_3_-HTCC prepared by hydrothermal treatment via glucose, flour and starch; Figure S10: Time profiles and corresponding apparent rate constants for the photocatalytic Cr(VI) reduction over HTCC photocatalysts prepared by nitric acid assisted hydrothermal treatment of different precursors under irradiation; Figure S11: Photocatalytic Cr(VI) reduction of HNO_3_-HTCC under different pH conditions; Figure S12: Photocatalytic Cr(VI) reduction of HNO_3_-HTCC with different nitric acid concentrations; Figure S13: ESR spectra of HTCC, HNO_3_-HTCC and Na_2_S_2_O_8_-HTCC; Figure S14: ESR spectra of the DMPO-•O_2_^−^ for HTCC, HNO_3_-HTCC and Na_2_S_2_O_8_-HTCC under irradiation; Figure S15: Time profiles for the photocatalytic Cr(VI) reduction over HTCC (a,b), HNO_3_-HTCC (c,d) and Na_2_S_2_O_8_-HTC (e,f) in the presence of monochloroacetic acid or nitrite with different concentrations; Figure S16: The cyclic tests for the photocatalytic Cr(VI) reduction by HNO_3_-HTCC; Table S1: The ratio of region I/II in FT-IR spectra of HTCC samples; Table S2: Elemental composition of HTCC samples; Table S3: Rate constants of radical scavenger with hydrogen radicals (H•) and hydrated electrons (e_aq_^−^). Refs. [29,34,35] are cited in the Supplementary Materials.
