# Supplementary material for: Highly Efficient Photocatalysis Towards Synthesis of Crystalline Hydrothermal Carbonation Carbon
Source: Molecules. 2026 Apr 25;31(9):1421. doi: 10.3390/molecules31091421 (PMC13165260; doi:10.3390/molecules31091421)
Supplement: Supplementary file 1 [file molecules-31-01421-s001.zip › molecules-4238614-supplementary.pdf]

Electronic Supplementary Information

Highly efficient photocatalysis towards synthesis of crystalline hydrothermal carbonation carbon

Xunxian Chen [1], Yu Luo [2], Zihang Zhang [2] Yingming Chen [2] and Zhen Wan [2]*

[1] Foshan Water Industry Group Co., Foshan 528000, China;

[2] Key Laboratory of Catalysis and Materials Science of the State Ethnic Affairs Commission and Ministry of Education, College of Resources and Environmental Science, South-Central Minzu University, Wuhan 43007, China;

* Correspondence: 2018030@scuec.edu.cn; Tel.: +86-27-67843990

Figures

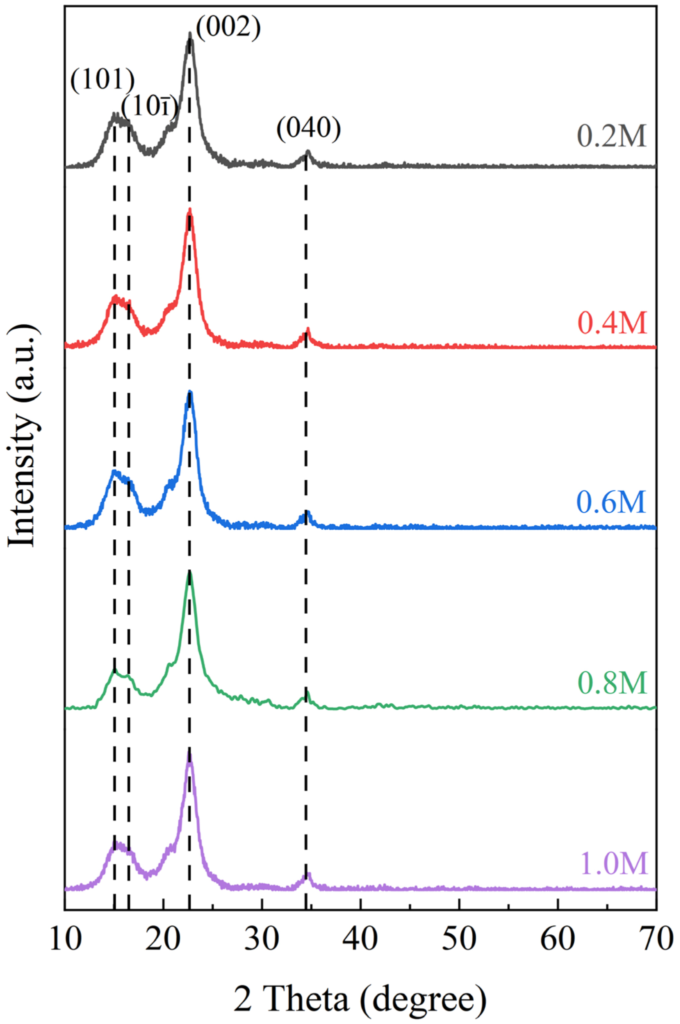

Figure S1. XRD patterns of HNO_3-HTCC prepared by hydrothermal treatment added with different concentrations of nitric acid.

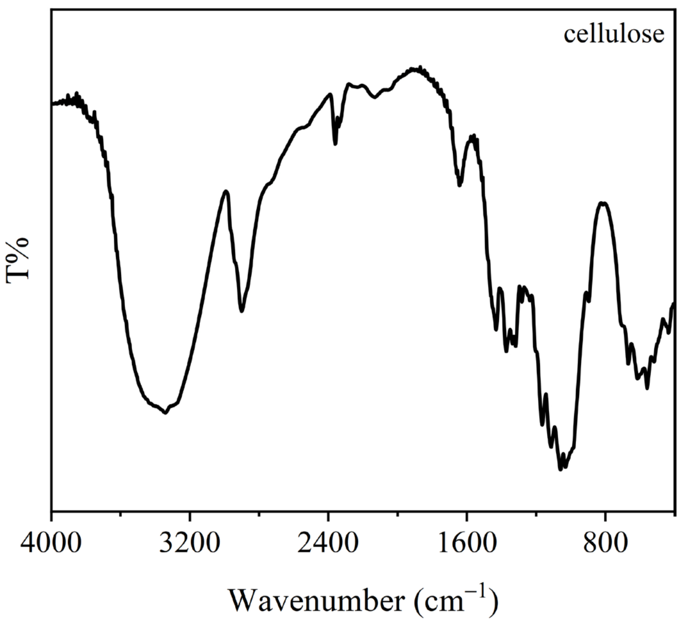

Figure S2. FT-IR spectrum of original cellulose.

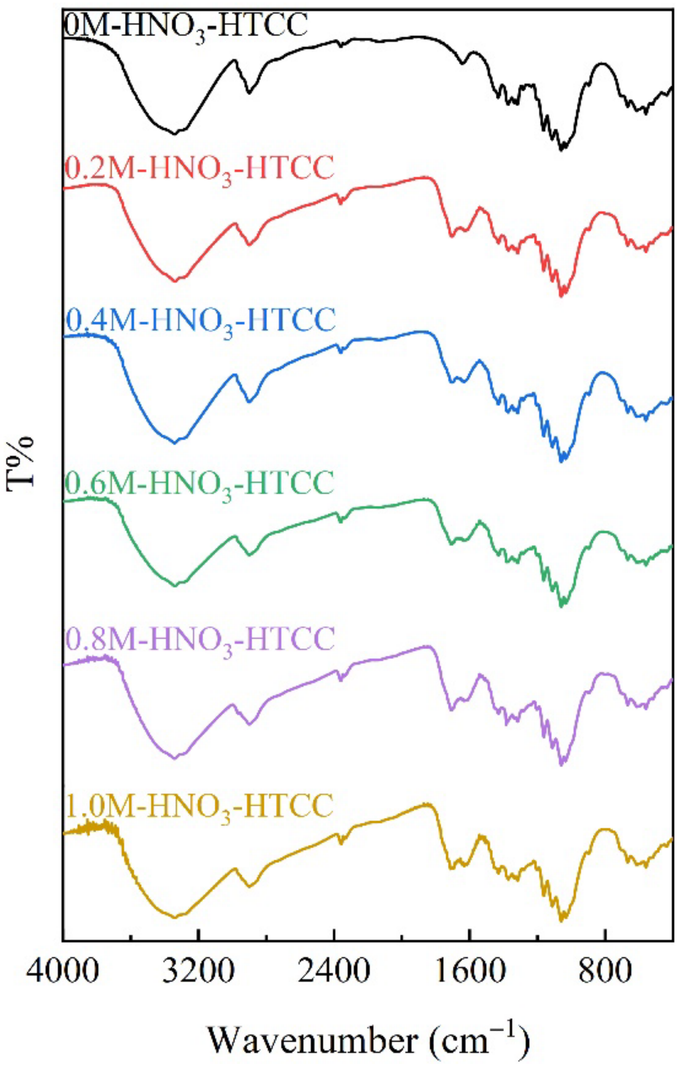

Figure S3. FT-IR spectra of HNO_3-HTCC prepared by hydrothermal treatment added with different concentrations of nitric acid.

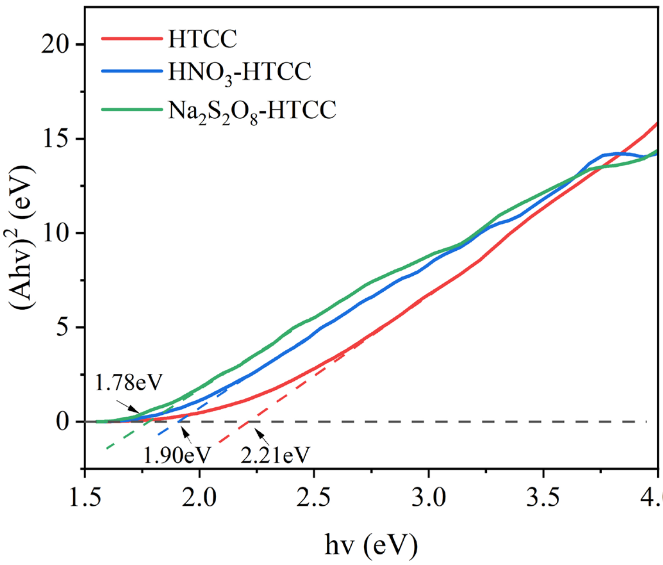

Figure S4. The Converted Kubelka-Munk plots of HTCC, HNO_3-HTCC and $Na_2S_2O_8$-HTCC. The band gap energy (Eg) of HTCC photocatalysts were obtained by converting the UV-vis diffuse reflection spectra via the classical Tauc approach, in which the Eg values of HTCC, HNO_3-HTCC and $Na_2S_2O_8$-HTCC were 2.21, 1.90 and 1.78 eV respectively.

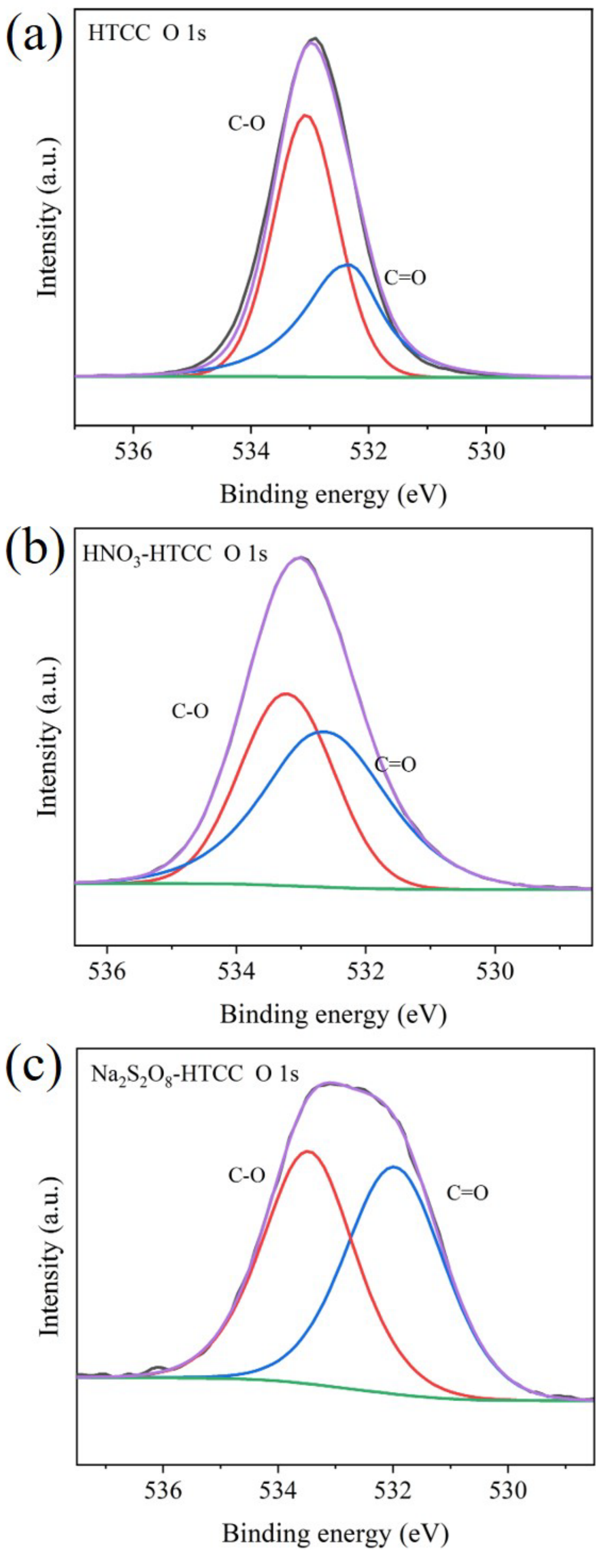

Figure S5. XPS O 1s spectra of the (a) HTCC, (b) HNO_3-HTCC and (c) $Na_2S_2O_8$-HTCC.

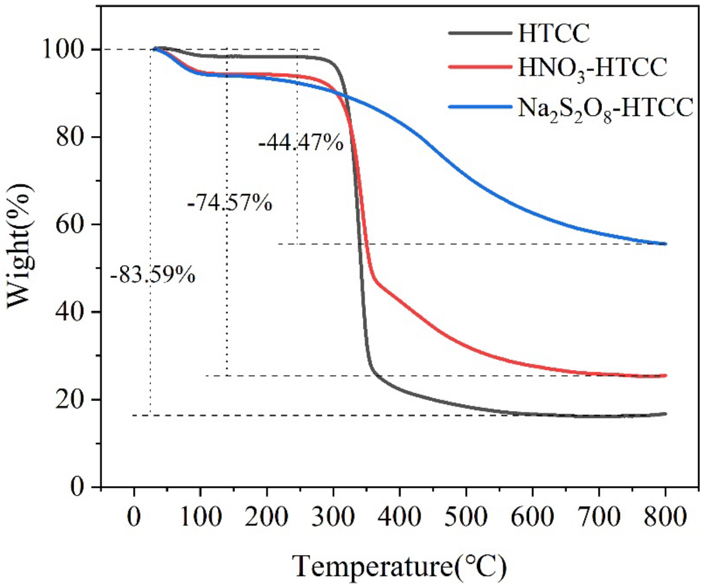

Figure S6. Thermogravimetry curves of HTCC, HNO_3-HTCC and $Na_2S_2O_8$-HTCC.

In Fig S6, the measurements were carried out in the atmosphere of nitrogen and all HTCC photocatalysts exhibited significant weight loss at the region of 300-800 °C. The total weight loss of HTCC, HNO_3-HTCC and $Na_2S_2O_8$-HTCC were 83.59 %, 74.57 % and 44.47 % respectively, indicating the reinforced dehydration in aromatization process of HNO_3-HTCC and $Na_2S_2O_8$-HTCC as compared to that of the HTCC.

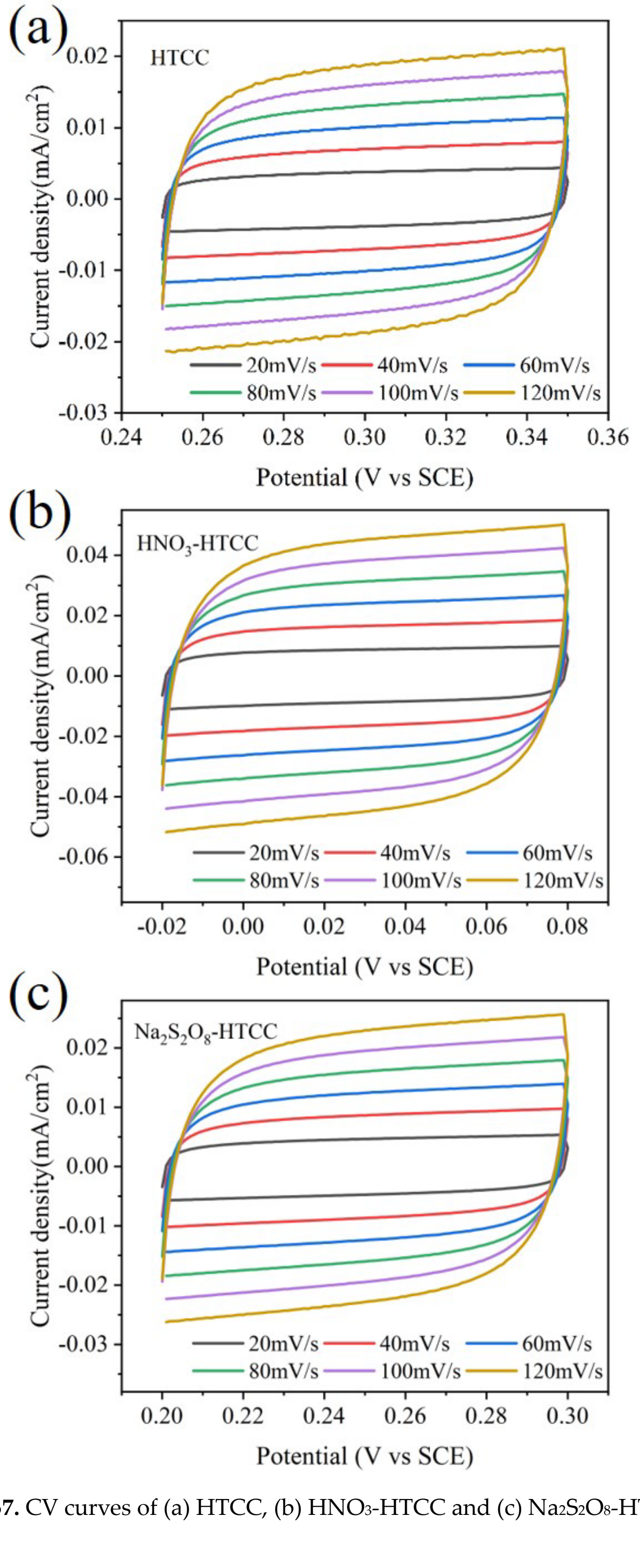

Figure S7. CV curves of (a) HTCC, (b) HNO_3-HTCC and (c) $Na_2S_2O_8$-HTCC.

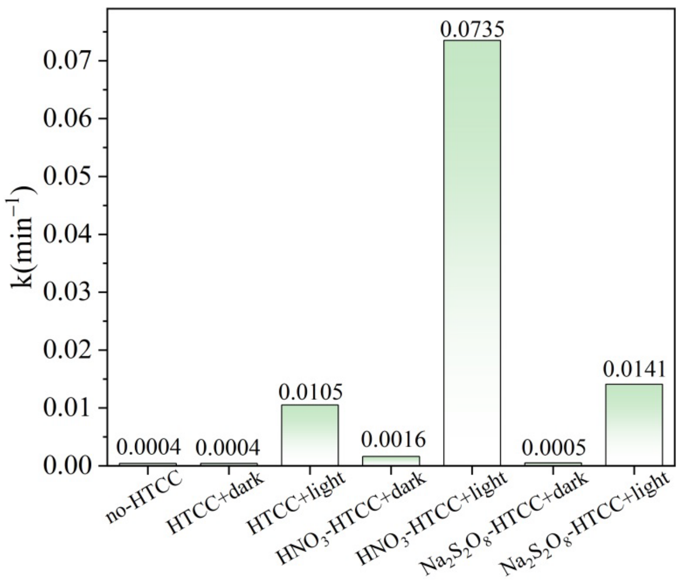

Figure S8. The apparent rate constants of HTCC, HNO_3-HTCC and $Na_2S_2O_8$-HTCC in the photocatalytic Cr(VI) reduction.

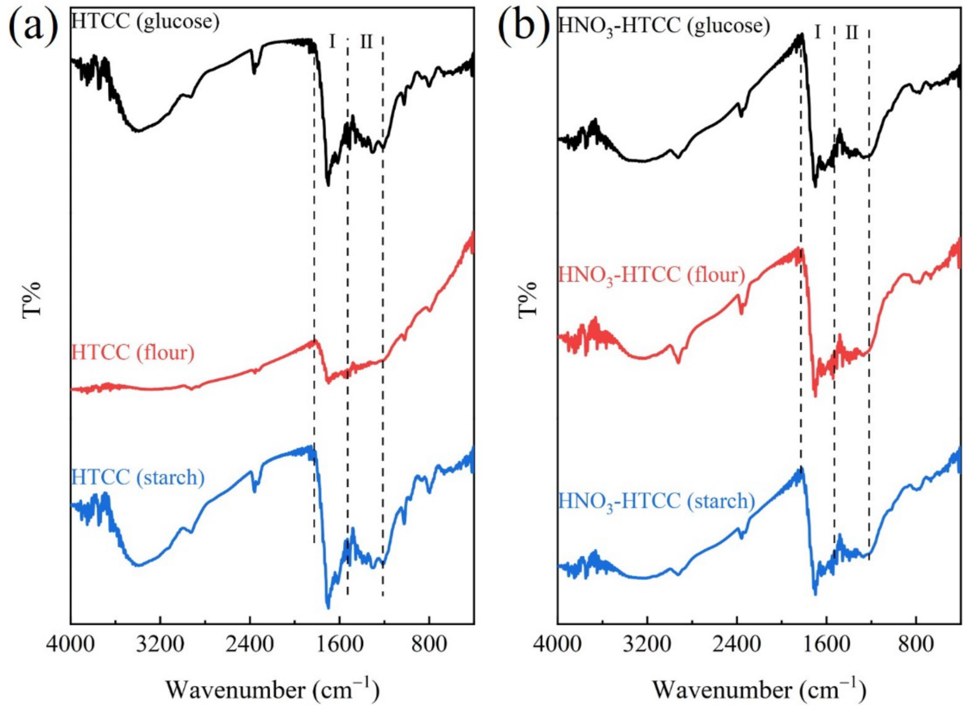

Figure S9. FT-IR spectra of (a) HTCC and (b) HNO_3-HTCC prepared by hydrothermal treatment via glucose, flour and starch.

As shown in Fig S9, compared with the HTCC prepared by glucose, flour and starch, their corresponding HNO_3-HTCC exhibited more intensive peak of sp^2 hybridized C=C stretching at 1600 cm^{-1}, indicating their higher aromatization level. As shown in Fig S10, the HNO_3-HTCC photocatalysts prepared by nitric acid-assisted hydrothermal treatment exhibited enhanced photocatalytic activities of Cr(VI) reduction than those without addition of nitric acid during the treatment. When the precursor is glucose, flour and starch, the photocatalytic activities of Cr(VI) reduction by these amorphous HNO_3-HTCC photocatalysts are about 2.5 times higher than of the HTCC photocatalysts. Meanwhile, the HNO_3-HTCC prepared by cellulose exhibited approximately seven-fold higher activity than HTCC, in which the improvement of activity is more significant. Apparently, the crystalline structure of HNO_3-HTCC played more critical roles in the photocatalytic reactions.

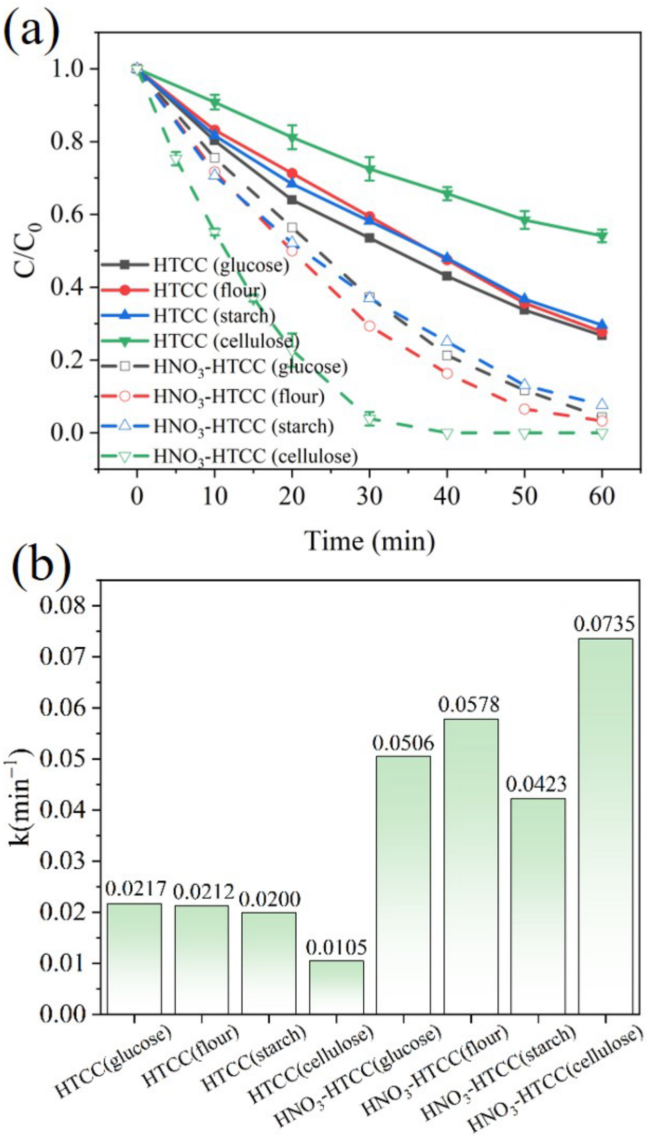

Figure S10. Time profiles and corresponding apparent rate constants for the photocatalytic Cr(VI) reduction over HTCC photocatalysts prepared by nitric acid assisted hydrothermal treatment of different precursors under irradiation.

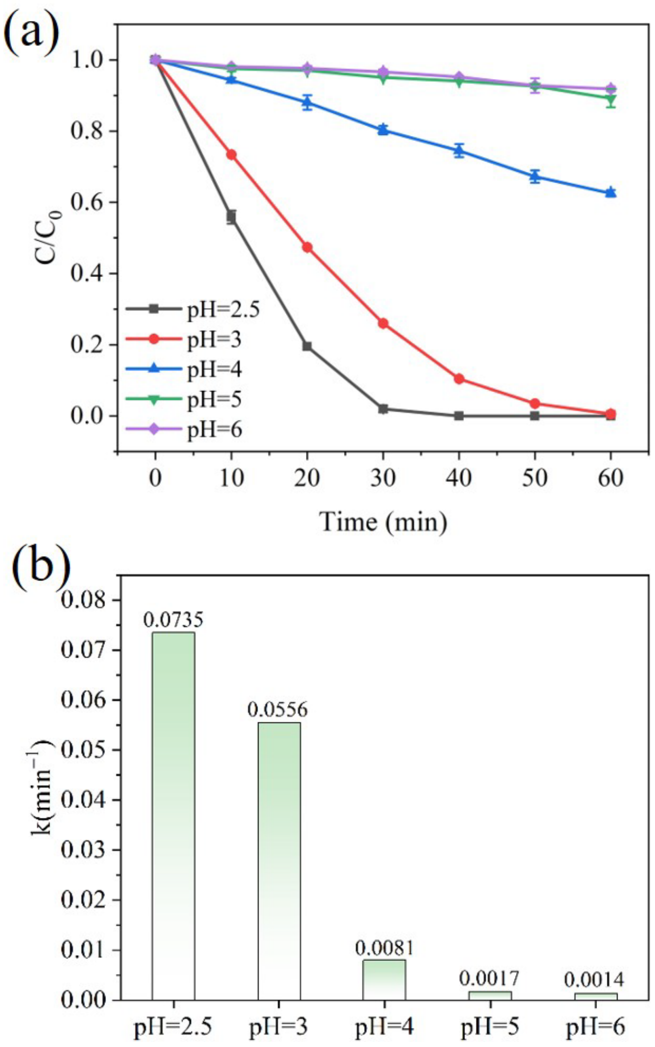

Figure S11. Photocatalytic Cr(VI) reduction of HNO_3-HTCC under different pH conditions.

As shown in Fig S11, the photocatalytic performance of the HNO_3-HTCC sample was found to be significantly influenced by the pH of the solution, exhibiting a gradual decline as the pH increased. This observed outcome can be ascribed to two points. On one hand, it was confirmed that the active hydrogen radicals (H•) were the dominant active species in the photocatalytic Cr(VI) reduction of HNO_3-HTCC. The generation of H• is closely related to the pH. On the other hand, according to Nernst equation, high proton concentration is favorable to the reduction of Cr (VI).

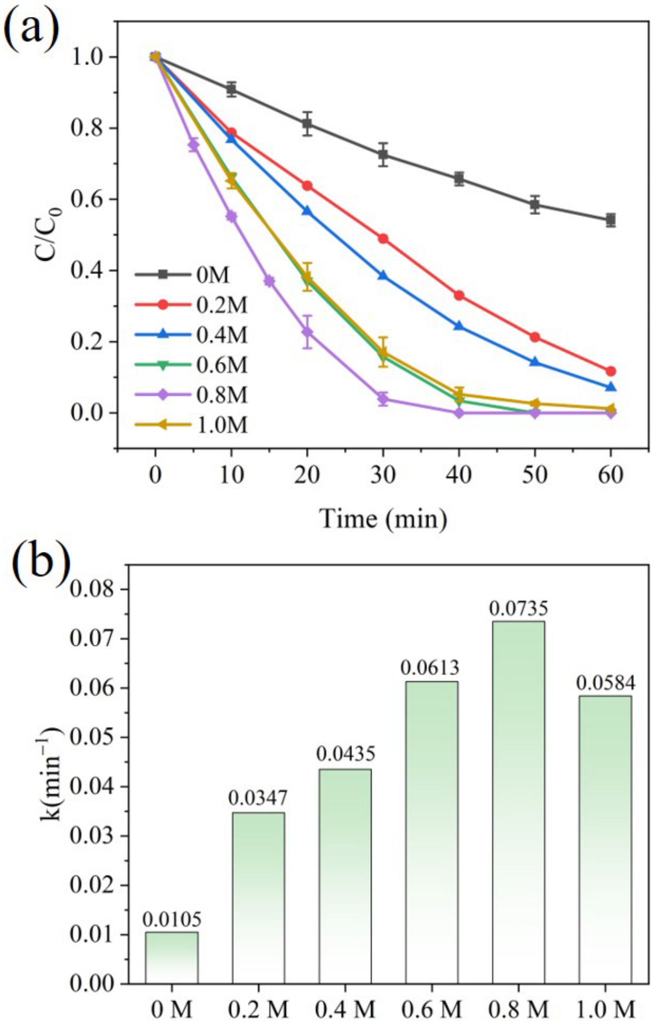

Figure S12. Photocatalytic Cr(VI) reduction of HNO_3-HTCC with different nitric acid concentrations.

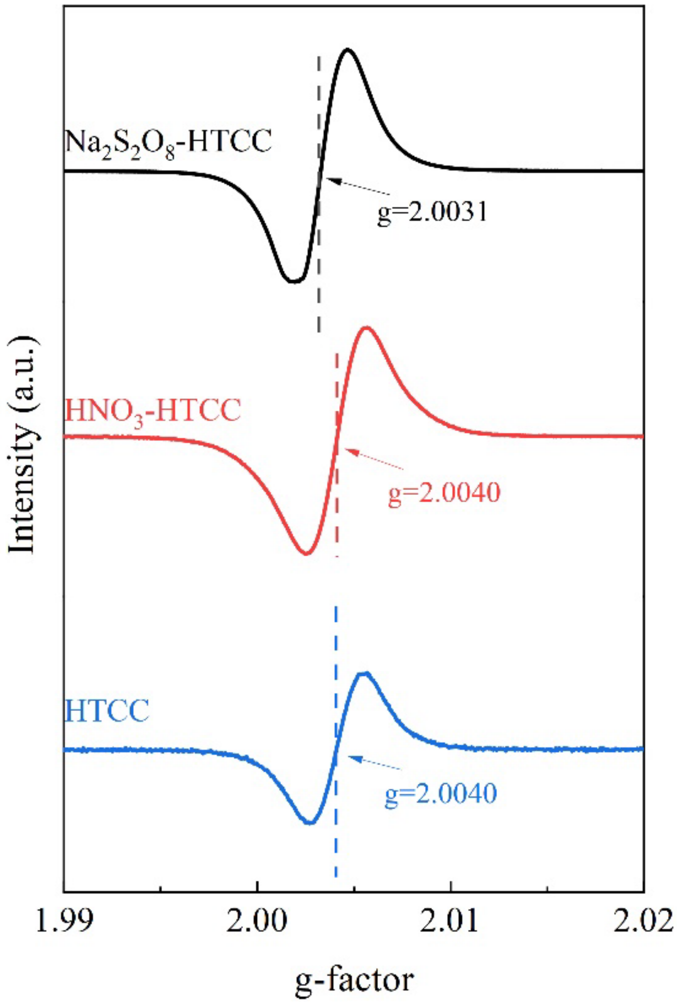

Figure S13. ESR spectra of HTCC, HNO_3-HTCC and $Na_2S_2O_8$-HTCC.

As shown in Fig S13, the ESR spectra of HTCC, HNO_3-HTCC and $Na_2S_2O_8$-HTCC indicated the existence of persistent free radicals (PFRs) on the surface of these HTCC photocatalysts. For carbon-centered PFRs, it was reported that the g-factor value of spectral splitting factor of ESR spectra was usually less than 2.0030 [29]. If the g-factor of carbon-centered PFRs is from 2.0030 to 2.0040, it might mean the existence of adjacent oxygen atoms. While the g-factor was larger than 2.0040, it indicated the PFRs were from an oxygen-centered situation.

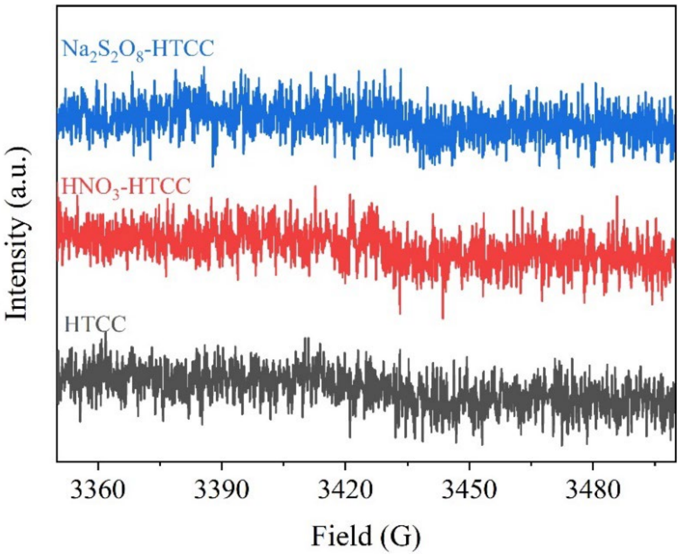

Figure S14. ESR spectra of the DMPO-•O_2^- for HTCC, HNO_3-HTCC and $Na_2S_2O_8$-HTCC under irradiation.

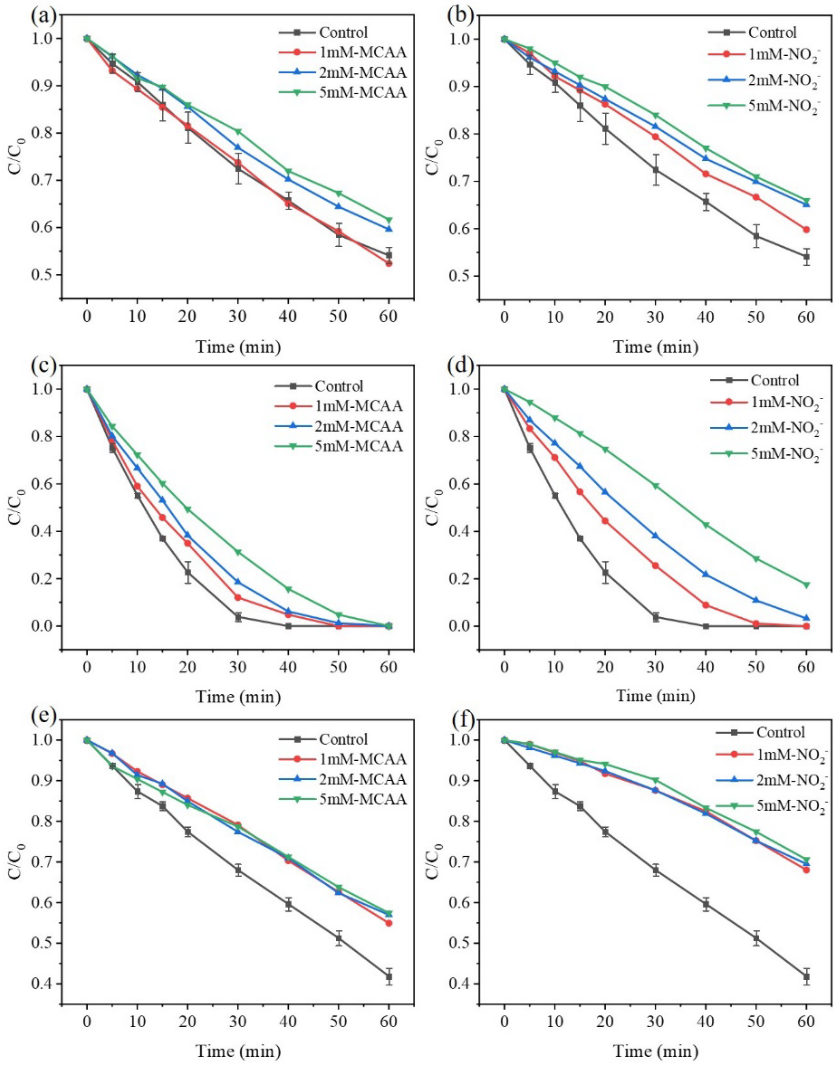

Figure S15. Time profiles for the photocatalytic Cr(VI) reduction over HTCC (a, b), HNO_3-HTCC (c, d) and $Na_2S_2O_8$-HTC (e, f) in the presence of monochloroacetic acid or nitrite with different concentrations.

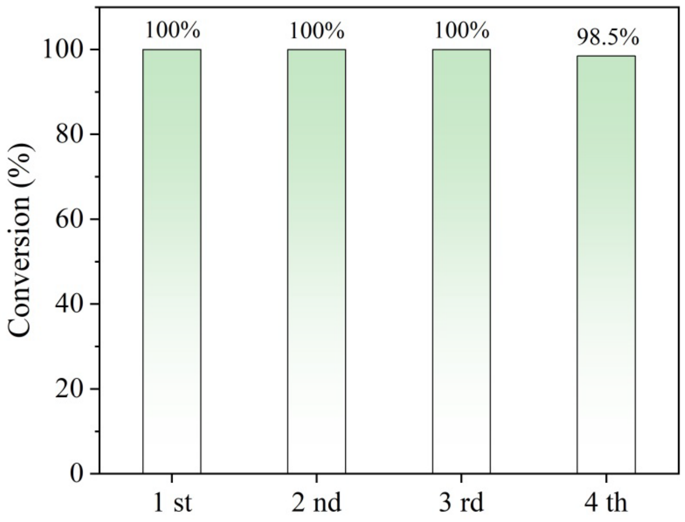

Figure S16. The cyclic tests for the photocatalytic Cr(VI) reduction by HNO_3-HTCC.

Tables

Table S1. The ratio of region I/II in FT-IR spectra of HTCC samples.

Sample	HTCC	HNO_3-HTCC	$Na_2S_2O_8$-HTCC
region I/II	0.116	0.787	1.116

Table S2. Elemental composition of HTCC samples.

Sample	Element content/Wt%			
	C	H	O	N
**HTCC**	40.54	7.17	51.09	0.12
**HNO_3-HTCC**	51.86	7.97	38.60	0.04
**$Na_2S_2O_8$-HTCC**	64.87	4.87	28.24	0

Table S3. Rate constants of radical scavenger with hydrogen radicals (H•) and hydrated electrons (e_{aq}^-).

Ions	Rate constants ($M^{-1}\,s^{-1}$)		Ref.
	·H	e_{aq}^-	
**NO_2^-**	7.1×10^8	3.5×10^9	[34, 35]
**MCAA**	6.5×10^3	6.9×10^9	[34, 35]
